# Prophylactic Palmitoylethanolamide Prolongs Survival and Decreases Detrimental Inflammation in Aged Mice With Bacterial Meningitis

**DOI:** 10.3389/fimmu.2018.02671

**Published:** 2018-11-16

**Authors:** Ev Christin Heide, Laura Bindila, Julia Maria Post, Dörthe Malzahn, Beat Lutz, Jana Seele, Roland Nau, Sandra Ribes

**Affiliations:** ^1^Institute of Neuropathology, University Medical Center Göttingen, Göttingen, Germany; ^2^Institute of Physiological Chemistry, University Medical Center of the Johannes Gutenberg University Mainz, Mainz, Germany; ^3^mzBiostatistics, Statistical Consultancy, Göttingen, Germany; ^4^Department of Geriatrics, Evangelisches Krankenhaus Göttingen-Weende, Göttingen, Germany

**Keywords:** endocannabinoid, immunomodulation, aging, lipidomics, antimicrobial stewardship, microglia, *Escherichia coli*

## Abstract

Easy-to-achieve interventions to promote healthy longevity are desired to diminish the incidence and severity of infections, as well as associated disability upon recovery. The dietary supplement palmitoylethanolamide (PEA) exerts anti-inflammatory and neuroprotective properties. Here, we investigated the effect of prophylactic PEA on the early immune response, clinical course, and survival of old mice after intracerebral *E. coli* K1 infection. Nineteen-month-old wild type mice were treated intraperitoneally with two doses of either 0.1 mg PEA/kg in 250 μl vehicle solution (*n* = 19) or with 250 μl vehicle solution only as controls (*n* = 19), 12 h and 30 min prior to intracerebral *E. coli* K1 infection. The intraperitoneal route was chosen to reduce distress in mice and to ensure exact dosing. Survival time, bacterial loads in cerebellum, blood, spleen, liver, and microglia counts and activation scores in the brain were evaluated. We measured the levels of IL-1β, IL-6, MIP-1α, and CXCL1 in cerebellum and spleen, as well as of bioactive lipids in serum in PEA- and vehicle-treated animals 24 h after infection. In the absence of antibiotic therapy, the median survival time of PEA-pre-treated infected mice was prolonged by 18 h compared to mice of the vehicle-pre-treated infected group (*P* = 0.031). PEA prophylaxis delayed the onset of clinical symptoms (*P* = 0.037). This protective effect was associated with lower bacterial loads in the spleen, liver, and blood compared to those of vehicle-injected animals (*P* ≤ 0.037). PEA-pre-treated animals showed diminished levels of pro-inflammatory cytokines and chemokines in spleen 24 h after infection, as well as reduced serum concentrations of arachidonic acid and of one of its metabolites, 20-hydroxyeicosatetraenoic acid. In the brain, prophylactic PEA tended to reduce bacterial titers and attenuated microglial activation in aged infected animals (*P* = 0.042). Our findings suggest that prophylactic PEA can counteract infection associated detrimental responses in old animals. Accordingly, PEA treatment slowed the onset of infection symptoms and prolonged the survival of old infected mice. In a clinical setting, prophylactic administration of PEA might extend the potential therapeutic window where antibiotic therapy can be initiated to rescue elderly patients.

## Introduction

Aging is a natural process accompanied by a progressive deterioration of physiological and cognitive functions (1). The age-related decline of the immune function is responsible for reduced vaccine responses and increased incidence and severity of infections in elderly persons (2, 3). For example, the mortality rate of acute bacterial meningitis is 1.5–3 times higher in individuals >65 years than in young adults (4–7). Elderly persons frequently consume immunosuppressive drugs such as corticoids, which in turn aggravates their immunodeficiency (8). Moreover, atypical clinical manifestations complicate the correct diagnosis of infections and delay the start of antibiotic therapy in the geriatric patient, contributing to an adverse outcome (4, 5). The risk for complications arising from treatment delay may increase by up to 30% per hour (9). To optimize the treatment efficiency, the German Society of Neurology recommends the initiation of antibiotic therapy within 1 h after admission and immediately after sampling of blood cultures and lumbar puncture (LP). When LP is delayed for any reason, antibiotics shall be administered prior to LP (10). When antibiotic therapy is started after the point of no return, the patient will die irrespective of the therapy chosen (11). Accordingly, new avenues to prevent the emergence of infections and to extend the therapeutic window for potential antibiotic therapy in the elderly population are required.

Nutritional interventions have been broadly used to boost immunity in the aged population (12). Besides their potential in promoting healthy longevity, supplements strengthening the immune defense constitute a complementary approach to reduce and improve antibiotic use (and to diminish the risk of antibiotic resistance) in the elderly population, especially in those living in long-term care facilities (LTCFs) (13). Endogenous and dietary lipids such as palmitoylethanolamide (PEA) contribute to the maintenance of homeostasis including modulation of the immune response (14). We have shown that exogenous PEA promotes pathogen uptake by microglial cells in young animals without the concomitant release of pro-inflammatory mediators that contribute to brain damage in bacterial meninigitis (15). During aging, senescent dysfunctional microglia acquire a chronic pro-inflammatory phenotype (16, 17). Primed/reactive microglial cells amplify and prolong inflammation upon infection, and exacerbate neurocognitive disability in elderly patients (18). According to the immunocompromised status of the elderly, aged murine microglia showed impaired bacterial clearance compared to young cells (19). Immunomodulation to strengthen the immune response against infections, including microglial function, is therefore clinically relevant in geroscience (20).

PEA acts as a lipid messenger. Besides being a dietary supplement, PEA is also abundantly synthesized in the mammalian brain (21). PEA is considered an endocannabinoid (eCB)-like compound; it shares with the endogenous eCB anandamide (AEA) enzymes involved in their biosynthesis and degradation, and can act on similar targets (22). The eCB system regulates the body's inflammatory response, and particularly in the central nervous system (CNS), it modulates microglial activation. Chronic infusion of an agonist of cannabinoid receptors (CBs) 1 and 2 increased neurogenesis, reduced the pro-inflammatory response in the brain, and improved memory in aged rats (23, 24). However, using compounds that target neuronal CB1 entails the risk of collateral undesired psychoactive effects (25). PEA does not bind to CB1/CB2, thus does not possess psychotropic activity (26), rendering it an attractive therapeutic agent. Other bioactive lipids, such as arachidonic acid (AA, an intermediate product of the metabolism of AEA and 2-arachidonoylglycerol [2-AG]) and eicosanoids (eiCs, resulting metabolites of the oxidation of AA), are also key effectors in neuroinflammation (27–29).

PEA as nutraceutical has been used to successfully treat over 3,000 patients for neuropathic pain, chronic inflammation, and inflammatory bowel disease (30, 31). PEA showed a neuroprotective role in several animal models of neurodegeneration and attenuated neuroinflammation in a mouse model of epilepsy (32). Here, we aimed to study the immunomodulatory potential and life-prolonging effect of prophylactic exogenous PEA in aged mice after intracerebral *E. coli* infection without antibiotic treatment. We investigated the potential of PEA to reduce bacterial spread, attenuate the release of pro-inflammatory cytokines and chemokines, and diminish the levels of AA and the subsequent production of eiCs.

## Materials and methods

### Bacteria

The *E. coli* strain K1 (serotype O18:K1:H7) originally isolated from a child with meningitis was used in all experiments (33). Bacteria were grown over-night on blood agar plates, harvested in 0.9% saline and stored at −80°C. All experiments were performed with aliquots of the same bacterial culture stored at −80°C. In each experiment one of these aliquots was thawed and diluted in saline to the required bacterial concentration.

### *In-vitro* growth curves assessing the effect of PEA on bacterial growth

Freshly prepared bacteria from a cryo-conserved aliquot were added to different tubes containing brain heart infusion and (i) PEA 0.01 μg/ml, (ii) PEA 0.1 μg/ml, (iii) PEA 1 μg/ml, and (iv) 0.017% DMSO (same amount of DMSO as in tubes with PEA 0.01, 0.1, and 1 μg/ml). Each condition was tested in triplicates. Tubes were incubated at 37°C under rotation (100 rpm). At different time points, the number of viable bacteria was assessed by performing 10-fold dilutions in 0.9% NaCl and plating on sheep blood agar. Viable bacteria were quantified after overnight incubation at 37°C.

### Experimental design

All procedures were reviewed and approved by the Animal Care Committees at the University Medical Hospital of Göttingen and at the government of Lower Saxony, Germany. Thirty-eight aged C57Bl/6 mice (18–19 months old, Janvier, France) were divided in two groups, one treated with PEA (*n* = 19) and the other with vehicle solution (*n* = 19), and later infected. Moreover, 8 non-infected mice were sacrificed 24.5 h after injection of vehicle solution to assess the morphology of microglia in aged healthy mice. PEA (0.1 mg/kg in 250 μl 0.9% saline containing 0.47–0.55% dimethyl sulfoxide [DMSO] according to the body mass) and vehicle (250 μl 0.9% saline containing 0.47–0.55% DMSO) were administered intraperitoneally 12 h and 30 min prior to the induction of meningoencephalitis defined as time point 0 h. The intraperitoneal administration route of PEA was chosen to reduce distress in mice and to ensure exact dosing. Previous studies have demonstrated the neuroprotective properties of intraperitoneal (ip) PEA (34, 35). The animals were anesthetized by ip injection of 2 mg ketamine and 0.2 mg xylazine. Then, meningitis was induced by the injection of 10 μl 0.9% saline containing *E. coli* K1 into the superficial right frontal cortex and subarachnoid space through the right fronto-lateral skull (19, 33). Animals were weighed and scored daily as previously reported (19, 33), and euthanized when they showed severe lethargy and impaired mobility (clinical score [CS] = 3), or presented a decrease of ≥20% of the initial body weight. None of the animals died spontaneously. Mice were housed in groups of up to 4 per cage in a 12 h light/dark cycle with full access to food and water. No sample or outlier value was excluded. Clinical scoring, weighing, measurement of the mediators of inflammation, and bioactive lipids, and histological evaluation were done in a blinded way.

In survival studies, PEA- and vehicle-treated animals received 0.8 × 10^3^ colony-forming units (CFU) *E. coli*/mouse, and were followed-up for 14 days after infection (*n* = 9/group). Differences in the course of the disease were analyzed by Kaplan–Meier curves of overall survival and symptom-free (CS = 0, no apparent behavioral abnormality) survival times. In short-term experiments (*n* = 10/group), PEA- and vehicle-injected mice were challenged with 3 × 10^3^ CFU E. coli/mouse and were sacrificed 24 h after infection. Non-infected mice were sacrificed 24.5 h after the second vehicle administration to allow comparisons with infected animals. Survival studies and short-term experiments were performed twice, respectively.

### Sample processing

Blood was obtained in anesthetized mice (same doses as described above) by cardiac puncture (up to 0.6–0.9 ml blood/mouse according to body mass). Death was confirmed by cervical dislocation. Ten microliters of blood were serially diluted in 0.9% saline for the determination of bacterial concentrations. The rest was stored at 4°C for 30 min and then centrifuged at 3,000 × g for 10 min at 4°C. Serum was used for quantification of the levels of AA and eiCs. The brain, spleen, and liver were isolated. The cerebellum was manually dissected from the brain. The left half of the cerebellum, half of the spleen, and liver were weighed, immediately diluted in 0.9% NaCl at a ratio of 1:10 for cerebellum, 1:5 for spleen, and 1:2 for liver, respectively to then be homogenized using a sterile micropestle. For the direct quantification of bacteria, 1:10 dilutions were performed and plated on blood agar. The rest of cerebellar/splenic homogenates were stored at −20°C to assay cyto-/chemokine concentrations. We measured bacteria and inflammatory mediators in the cerebellum, because of the higher ratio of meninges/brain tissue compared with the neocortex. The cerebrum was fixed in 4% paraformaldehyde and used for histology.

### Targeted quantification of endogenous lipids

Serum levels of PEA and AA, as well as of representative eiCs generated from oxidation of AA via two major metabolic pathways were determined 24 h after infection: prostaglandin E_2_ (PGE_2_) as product of cyclooxygenase (COX)-2, and 20-hydroxyeicosatetraenoic acid [20 (S)-HETE] obtained upon cytochrome P450 (CYP) epoxygenases. PEA, AA, and eiCs were co-extracted using a previously developed and described liquid-liquid extraction method (36). Briefly, following animal sacrifice, blood, brain, and peripheral organs were sampled in this order. Seventy-five microliters of the serum prepared as described above (see Sample Processing section) were used for the extraction of lipids. The serum samples were allowed to quickly thaw on ice. Then ice-cold ethylacetate/n-hexane (9:1, v/v) containing deuterated standards of the targeted eiCs, PEA, and AA, respectively, followed by 0.1 M formic acid were added. Samples were then vortexed for 2 min, centrifuged for 20 min (13,000 rpm, 4°C) and then frozen for 10 min. The upper phase containing the lipids was recovered, evaporated to dryness, and reconstituted in 50 μl of acetonitrile/water (1:1, v/v) for quantitative analysis. The quantification of the lipid levels was carried out by liquid chromatography-multiple reaction monitoring (LC-MRM) using a targeted, multiplex assay involving positive and negative ion mode switching to achieve analysis of these compounds in a single run. All sample processing and extraction steps were carried out at 4°C to minimize *ex-vivo* alteration of lipid levels, and the time course from sampling to analysis was maintained similar for all animals in order to reduce sample variability sources, hence ensure reliable comparative study (36, 37).

### Cyto-/chemokine measurements

Pro-inflammatory cytokines (interleukin [IL]-1β, IL-6) and pro-inflammatory chemokines (macrophage inflammatory protein-1α [MIP-1α], chemokine [C-X-C-motif] ligand 1 [CXCL1]) were quantified by DuoSet enzyme linked immunosorbent assay Development kits in cerebellar and splenic homogenates of infected old animals. For all tested compounds, the sensitivity was 37.5 pg/g in the spleen and 75.0 pg/g in the cerebellum.

### Histological analyses

Paraffin-embedded, 2-μm coronal brain sections were used to visualize microglia by staining of ionized calcium-binding adapter molecule 1 (Iba-1) with the rabbit anti-Iba-1 polyclonal antibody (Wako Chemicals) (33). The number of Iba-1^+^ cells was determined in six different neocortical regions and the hippocampal fissure and then divided by the number of scored regions by a blinded observer. The Iba-1 staining revealed four divergent cell morphologies according to gradual steps of microglial activation (38, 39). Based on the most abundant morphology at a magnification of 20, a microglia activation score (AS) was given to each scored brain region in a blinded way. Microglial activation is a multi-step process that starts with hyper-ramification and subsequent enlargement of the cell body and more pronounced thickness and gradual retraction of the ramifications until acquisition of an ameboid morphology (38). Microglia with relative big somata but fine ramifications were scored as an AS of 1 (40). An AS of 2 was given to hypertrophic cells with thicker branches, while AS3 and AS4 were assigned to bushy and ameboid cells, respectively (39).

### Statistical analyses

Overall and symptom-free survivals were compared using the log-rank test. Other comparisons between PEA and vehicle groups were performed by the Mann–Whitney *U*-test. Correlations between (i) bacterial titers and microglial activation scores, and (ii) bacterial titers and cytokine/chemokine levels were analyzed using the Spearman's rank correlation coefficient (r_s_). The levels of pro-inflammatory cytokines (joint test on IL-1β and IL-6) and pro-inflammatory chemokines (joint test on MIP-1α and CXCL1) were analyzed by multivariate rank-sum tests in the spleen and cerebellum, respectively (41).

For all analyses, GraphPad Prism (version 5; GraphPad Software) and R software (version 3.2.2; www.cran.r-project.org) were used. Adjustment of the *p*-values for multiple comparisons in the same organ was performed by the Holm–Bonferroni method. Differences between two groups were considered statistically significant at *P* < 0.05.

## Results

### Prophylactic PEA delays onset of clinical symptoms and prolongs survival in old mice

**Figure 1:**
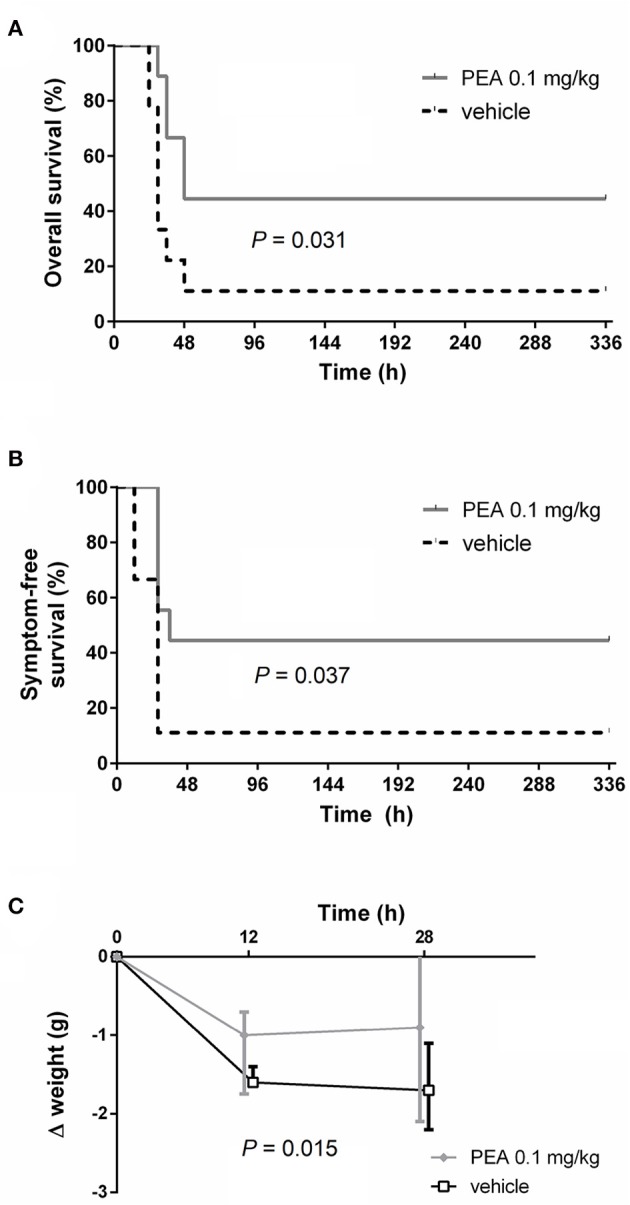
PEA prophylaxis delays the onset of symptoms and improves outcomes of old animals with *E. coli* meningoencephalitis. **(A)** Kaplan-Meier overall survival and **(B)** symptom-free survival curves after the intracerebral injection of 800 CFU *E. coli* K1/mouse in aged mice (*n* = 9/group, data from two independent experiments). **(C)** Median body weight loss until the first mouse died (28 h p.i.). Data from two independent experiments are shown as medians ± interquartile range (25th/75th percentile). Statistic analyses in **(A,B)** were performed by using the log-rank test, and in **(C)** by using the Mann–Whitney *U*-test after calculation of the individual AUCs of the weight loss 0–28 h p.i.

Aged mice receiving two ip doses of PEA (0.1 mg/kg) prior to infection exhibited longer survival after intracerebral injection of *E. coli* compared to vehicle-treated mice (median survival time 48 vs. 30 h; *P* = 0.031, log-rank test; *n* = 9/group; Figure 1A). Mortality rate assessed 14 days after inoculation was lower in animals receiving PEA than in controls without reaching statistical significance due to the relatively small group number (55.6% [5/9] vs. 88.9% [8/9]; *P* = 0.29, Fisher's exact test). PEA-injected animals displayed the first symptoms of disease (CS = 1) later than animals treated with vehicle (median symptom-free survival time 36 vs. 28 h; *P* = 0.037, log-rank test; Figure 1B). Body weight loss within 28 h post-infection (p.i.) was significantly lower in the PEA pre-treated group compared to the vehicle group (*P* = 0.015, comparison of the areas under the curves [AUCs] of the individual weight loss 0–28 h p.i. by Mann–Whitney *U*-test; Figure 1C).

### PEA significantly diminishes bacterial load in the bloodstream, spleen, and liver and tends to reduce bacterial concentrations in the brain

**Figure 2:**
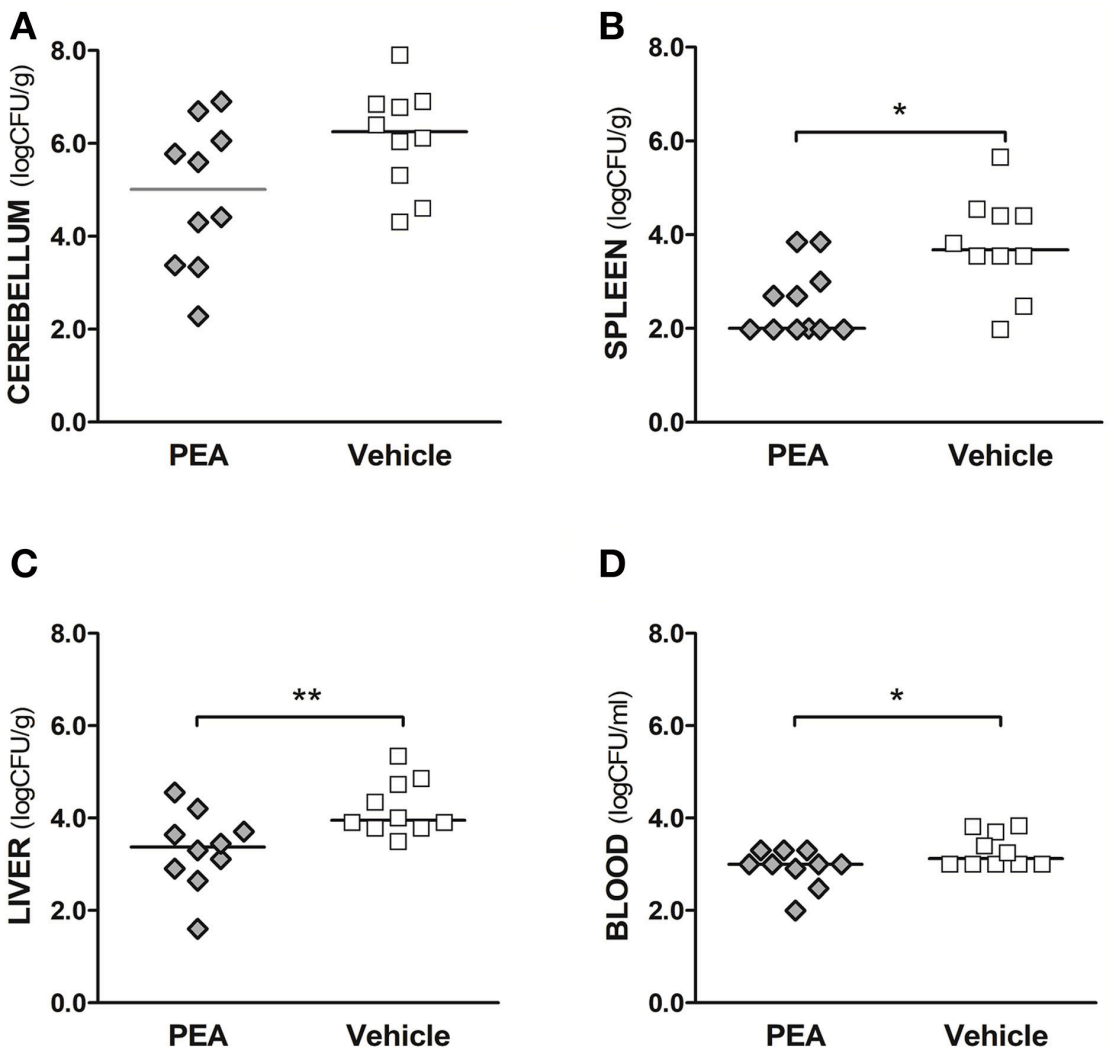
Exogenous PEA reduces bacterial spread in aged mice after the induction of *E. coli* K1 meningoencephalitis. Concentrations of *E. coli* K1 were quantified in **(A)** cerebellum, **(B)** spleen, and **(C)** liver (as log_10_CFU/g), as well as in **(D)** blood (as log_10_CFU/ml) of animals sacrificed 24 h after infection with 3,000 CFU *E. coli* K1/mouse (*n*= 10 mice/group, data from two independent experiments). Each symbol represents an individual mouse and bars indicate median values. ^**^P < 0.01, ^*^P < 0.05 between PEA and vehicle groups, using the Mann–Whitney *U*-test.

**Figure 3:**
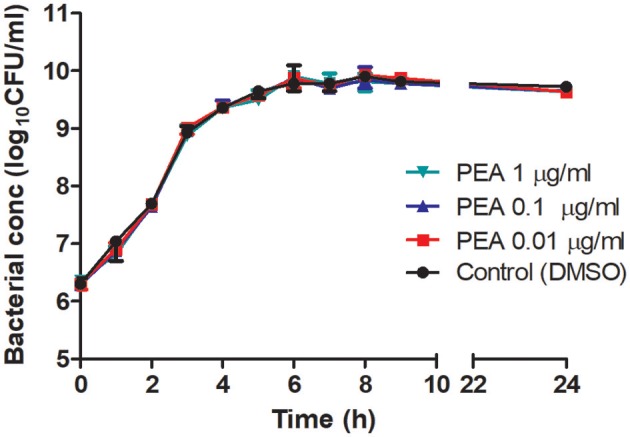
Growth curves of *E. coli* K1 in brain heart infusion in the presence of different concentrations of PEA and control medium without PEA (0.17% DMSO). At different time points, serial dilutions were plated on blood agar and viable bacteria (CFU/ml) were quantified after overnight incubation. Data are presented as median ± range (*n* = 3/condition).

We examined next whether PEA-driven protection was related to restricted pathogen growth in the brain and reduced spread of bacteria into the systemic circulation. For this, bacterial concentrations in cerebellum, spleen, and liver, as well as in blood samples, were determined in aged mice 24 h post-infection (*n* = 10/group, Figure 2). Bacteremia was significantly less severe in PEA-treated mice compared to control animals (*P* = 0.037, Mann–Whitney *U*-test). In PEA-treated animals bacterial concentrations were also significantly decreased in the spleen (*P* = 0.018, Mann–Whitney *U*-test) and liver (*P* = 0.006, Mann–Whitney *U*-test). Bacterial counts in the cerebellum tended to be lower in PEA-treated than in control animals (*P* = 0.065, Mann–Whitney *U*-test). We performed *in-vitro* growth curves with different concentrations of PEA to assess whether PEA showed antibacterial activity (Figure 3). No differences in the bacterial concentration-time curves were found, and therefore any direct antibacterial effect of PEA on *E. coli* K1 was excluded.

### PEA pre-treatment decreases the release of pro-inflammatory markers in the spleen of infected aged mice

**Figure 4:**
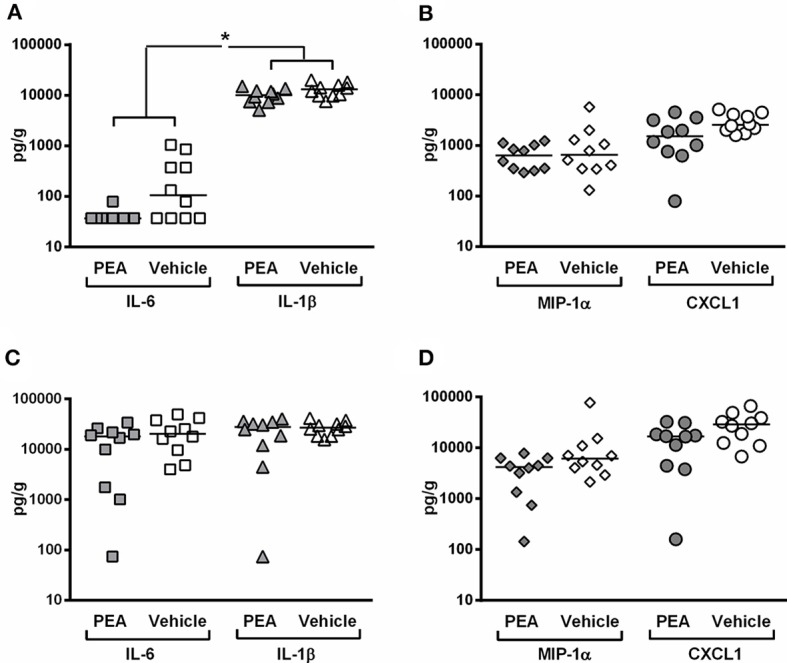
PEA pre-treatment attenuates inflammation in the spleen of aged animals at the early phase of meningitis. Concentrations of (pro-inflammatory cytokines interleukin (IL)-1β and IL-6, and pro-inflammatory chemokines macrophage inflammatory protein (MIP)-1α and C-X-C-motif ligand 1 (CXCL1) were measured in **(A,B)** spleen and **(C,D)** cerebellum from old infected animals pre-treated with PEA or vehicle (*n* = 10/group). The levels of cytokines and chemokines are expressed as pg/g of tissue. ^*^P < 0.05, examining whether PEA pre-treatment exerted a common effect on: (i) pro-inflammatory cytokines (joint analysis of IL-1β and IL-6) and (ii) chemokines (joint analysis of MIP-1α and CXCL1), using the multivariate rank-sum test with Holm-Bonferroni adjustment of the *P*-values for multiple comparisons in the same organ.

In the present study, bacterial concentrations in the spleen correlated with the levels of IL-6 (r_s_ = 0.45; *P* = 0.049), but not with the concentrations of IL-1β, MIP-1α, and CXCL1 (−0.05 ≤ r_s_ ≤ 0.09). The spleen of PEA-pre-treated animals had a decreased concentration of pro-inflammatory cytokines (*P* = 0.045, multivariate rank-sum test followed by Holm-Bonferroni correction; Figure 4A) with a significantly diminished IL-6 production (*P* = 0.038, Mann–Whitney *U*-test followed by Holm-Bonferroni correction). IL-6 levels were below the level of detection in 9 out of 10 PEA-treated infected animals. Moreover, prophylactic PEA tended to reduce chemokine release (*P* = 0.055, multivariate rank-sum test followed by Holm–Bonferroni correction; Figure 4B).

In the cerebellum, bacterial burden positively correlated with levels of IL-1β (r_s_ = 0.61; *P* = 0.004), IL-6 (r_s_ = 0.77; *P* < 0.0001), MIP-1α (r_s_ = 0.87; *P* < 0.0001), and CXCL1 (r_s_ = 0.84; *P* < 0.0001; *n* = 20). Consistent with a more effective bacterial elimination in cerebellum, animals pre-treated with PEA showed a trend toward lower chemokine levels compared to controls (*P* = 0.10, multivariate rank-sum test followed by Holm–Bonferroni correction, Figure 4D). Cytokine levels in cerebellum were comparable between PEA-treated and vehicle-treated animals (*P* = 0.38, multivariate rank-sum test followed by Holm–Bonferroni correction, Figure 4C).

### PEA attenuates microglial activation in infected aged mice

**Figure 5:**
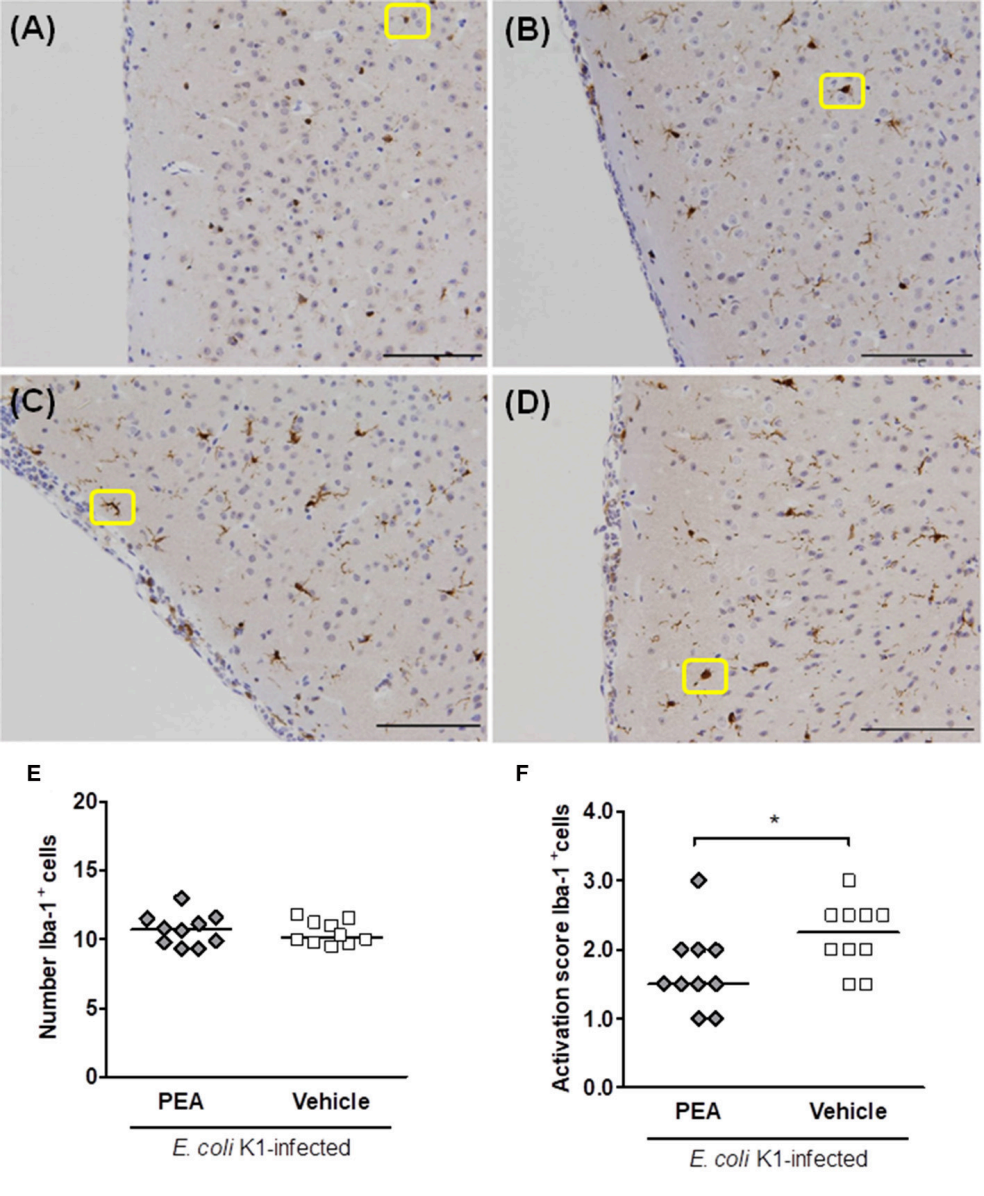
PEA prophylaxis does not modify microglial density, but attenuates microglial activation in aged infected mice. **(A–D)** Illustrative examples of the different cell morphologies (circled in yellow) and the corresponding activation scores in neocortex. A single activation score (AS) was given by a blinded investigator according to the most abundant cell morphology for each analyzed region: **(A)** microglia with small size and very fine ramifications (AS 1), **(B)** hypertrophic with thicker branches (AS 2), **(C)** bushy (AS 3), and **(D)** ameboid (AS 4); scale bars, 100 μm (magnification, ×20). **(E)** Number of Iba-1^+^ cells in brains of PEA- and vehicle-treated aged mice sacrificed 24 h after infection. **(F)** AS of Iba-1^+^ cells in brains from the same PEA- and vehicle-treated aged mice. In **(E,F)**, each symbol represents an individual mouse and bars indicate medians. ^*^P < 0.05, using the Mann–Whitney *U*-test.

We quantified Iba-1-stained cells in seven different brain regions of non-infected vehicle pre-treated and infected (24 h post-infection) PEA/vehicle-treated aged mice. Four different morphologies of Iba-1^+^ cells could be distinguished according to a gradual process of activation (38). Therefore, a blinded investigator assigned an activation score (AS) from 1 to 4 to ramified, hypertrophic, bushy, and ameboid cells, respectively. Non-infected aged mice showed uniformly distributed Iba-1^+^ cells with fine ramifications (AS 1, Figure 5A). Representative examples of activated hypertrophic, bushy, and ameboid Iba-1^+^ cells of infected old animals (AS 2–4, respectively) are shown in Figures 5B–D).

The quantification of Iba-1-stained microglial cells revealed equal numbers of microglia in PEA- and vehicle-treated groups 24 h post-infection (Figure 5E). However, the median score of microglial activation was significantly higher in vehicle-treated compared with PEA-injected infected old animals (Figure 5F, *P* = 0.042, Mann–Whitney *U*-test). PEA-treated infected aged animals mostly exhibited cells with a ramified-hypertrophic appearance (median AS [25th/75th percentile]: 1.5 [1.38/2.0]), while vehicle-injected animals more often showed a hypertrophic-bushy morphology (2.25 [1.88/2.5]). Furthermore, higher microglial activation scores tended to correlate with high concentrations of *E. coli* K1 in the brain (r_s_ = 0.42, *P* = 0.06; *n* = 20).

### PEA decreases AA levels and subsequent production of 20-HETE in serum of infected aged animals

**Figure 6:**
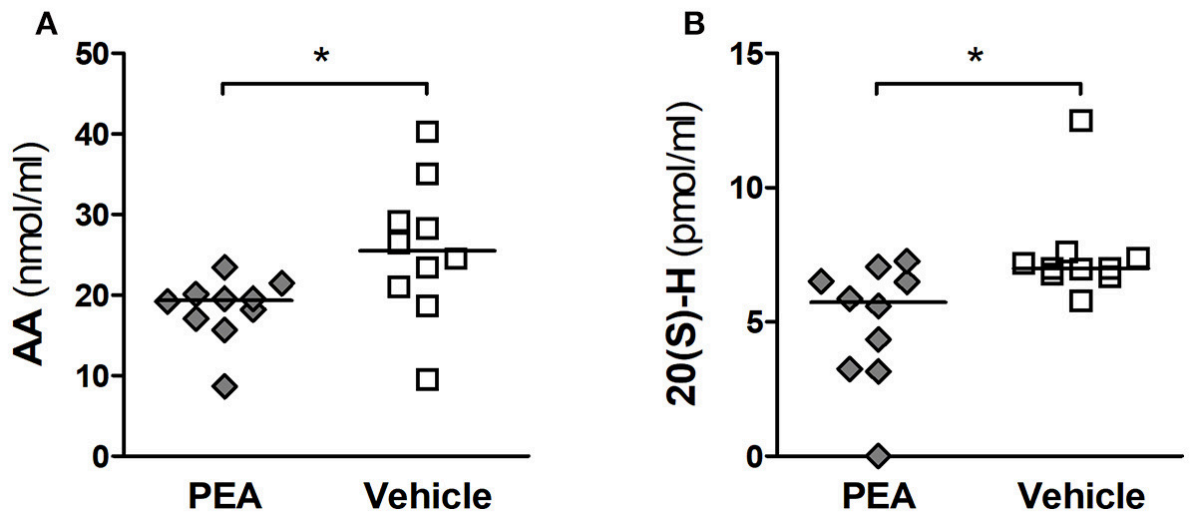
PEA reduces AA serum levels and its subsequent oxidation into 20-HETE in aged mice with meningitis. **(A)** Concentrations of arachidonic acid (AA, in nmol/ml), and **(B)** levels of 20-hydroxyeicosatetraenoic acid (HETE, in pmol/ml) were lower in the serum of aged infected animals pre-treated with PEA than in infected mice pre-treated with vehicle (*n* = 10/group). Serum levels of PEA and PGE_2_ were not significantly altered. ^*^P < 0.05 between PEA and vehicle groups, using the Mann–Whitney *U*-test followed by Holm–Bonferroni correction for multiple testing.

The levels of PEA, AA, PGE_2_, and 20-HETE in serum of PEA- and vehicle-pre-treated infected animals (*n* = 10/group) were assessed 24 h after infection. PEA pre-treatment decreased the concentrations of AA compared to vehicle (*P* = 0.036, Mann–Whitney *U*-test followed by Holm-Bonferroni correction, Figure 6A). We found no significant changes in PGE_2_, the selected end product of the COX pathway. Levels of PGE_2_ (median [25th/75th percentiles]) were 1.17 (0.75/2.89) pmol/ml in PEA-pre-treated, and 1.18 (1.04/2.39) nmol/ml in vehicle-administered old animals. Concentrations of PEA (median [25th/75th percentiles]) were 78.2 (53.3/112.8) pmol/ml in PEA-pre-treated and 89.8 (83.6/109.9) pmol/ml in vehicle-injected aged animals.

However, the concentrations of circulating 20-HETE (final product of the CYP450-mediated oxidation of AA) were significantly reduced in PEA-treated mice compared to vehicle (*P* = 0.032, Mann–Whitney *U*-test followed by Holm-Bonferroni correction, Figure 6B).

## Discussion

After the increased availability of vaccines to avert many cases of meningitis in children, priority was given to the development of preventive interventions in elderly persons, in whom the disease burden-associated fatality and disability rates remain dramatically high (4, 5). Specific nutritional interventions can reduce this risk and reverse some of the immune dysfunction associated with aging (12, 42, 43). PEA is available as a nutraceutical, but is also highly present in some foods such as egg yolk, soybean, soy lecithin, peanut oil, and alfalfa, and in smaller amounts in peas and tomatoes (44). In the 70s, six clinical trials enrolling children and young adults showed that PEA (under the brand name of *Impulsin*, administered up to 1,800 mg/d) decreased the incidence and severity of acute respiratory infections and influenza without reported side effects (45). Since then, no other trials have addressed the potential of PEA as an immunomodulatory agent in the setting of an infection.

In a recent report, we showed that PEA pre-treatment increased the survival rate and bacterial clearance of immunocompetent young mice challenged intracerebrally with *E. coli* (46). *In vitro* studies confirmed that PEA stimulates the phagocytosis of pathogens by macrophages and microglia (15, 47). Here, we demonstrated the efficacy of prophylactic PEA in prolonging the survival of aged infected animals in the absence of antibiotic treatment. At the early phase of infection, PEA-pre-treated mice showed lower bacterial titers in spleen, liver, and blood than vehicle-injected animals. To our knowledge, this is the first report documenting a life-prolonging and anti-bacterial effect of PEA in immunocompromised, old mice. The present study was performed in aged mice, which develop higher bacterial concentrations and a higher mortality compared to young adult mice after intracerebral infection with equal amounts of *E. coli* K1 (19). Because systemic complications are the leading cause of death in elderly patients with bacterial meningitis (7), reducing pathogen spread in the systemic circulation has the potential to broaden the therapeutic window, whereby the initiation of antibiotic therapies can rescue the patient, thus potentially improving survival of these patients in a clinical setting (11).

To identify possible mechanisms underlying PEA-induced protection in aged mice, we measured pro-inflammatory mediators such as cyto-/chemokines and certain bioactive lipids. Administration of exogenous PEA in old mice prior to infection effectively alleviated the excessive systemic release of pro-inflammatory mediators (IL-6, IL-1β, CXCL1, and MIP-1α), which are known to cause cerebral edema, vasculitis, and neuronal and axonal injury, leading to death or long-term sequelae in meningitis patients (48). Similarly, the administration of matrix metalloproteinase inhibitors in experimental meningococcal meningitis was accompanied by a reduction of pro-inflammatory mediators and attenuation of brain damage (49). In contrast to previous studies where a protective effect of PEA was confirmed at doses of 1–10 mg/kg (31, 50), here we show that also low doses of PEA (0.1 mg/kg) can prolong the life of old animals after infection.

As part of the host response against infection, lipid responses receive increasing attention in the scientific community (51). Little is known about how exogenous PEA can modulate other lipid mediators involved in neuroinflammation. Administration of exogenous PEA reduced COX-2 expression and PGE_2_ production in an animal model of epilepsy and colitis (32, 52). No data are available during infection. Here, we used targeted quantification of lipids to evaluate whether PEA could modify the levels of several bioactive lipids (51). In the systemic circulation, prophylactic PEA significantly reduced concentrations of two pro-inflammatory mediators: AA and one of its metabolites, 20-HETE which unveils a novel anti-inflammatory mode of action of PEA during infection. Because 20-HETE is a potent vasoconstrictor of brain microvessels that contributes to the development of vasospasm (53), the PEA-exerted effect on the production of this eiC is highly relevant in clinical practice. Cerebrovascular alterations as a result of vasculitis, vasospasm, or intra-arterial thrombosis are common complications occurring in one fourth of patients with meningitis and constitute a major risk factor for permanent neurological deficits and death, especially in geriatric patients (54). The serum levels of PEA were not elevated in the PEA pre-treated group, which may be due to the low concentration used in this study its quick metabolization and short half-life. In fact, after oral administration of PEA to rats (at dose of 100 mg/kg), the plasma concentration was highest after 15 min, and then dropped 2 h after administration to concentrations very close to the basal ones (44), while in mice peak plasma concentration were achieved 2.5 h after ip administration of 40 mg/kg PEA (32).

Microglial cells are key effectors in the resolution of CNS infections. Upon pathogen recognition, microglia undergo progressive morphological changes and accordingly acquire a plethora of new functions. Activated microglia show an increased soma and retraction of thickening processes and potentially augment the secretion of pro-inflammatory molecules (55). Brain aging is accompanied by a mild pro-inflammatory state that may increase basal microglial activation and worsen outcomes after infection (16–18). The ability of non-stimulated microglial cells from aged animals to phagocytose *E. coli* K1 was significantly impaired compared to cells isolated from young animals [median of 39.3 vs. 100%, (19)]. In the brain of our old infected animals, high bacterial loads correlated with increased microglial activation scores and an elevated release of inflammatory cytokines and chemokines. In the brain of PEA-pre-treated animals, a tendency toward reduced bacterial titers and diminished chemokine levels was observed. Concomitant to this reduced inflammatory response, microglia displayed significantly less morphological signs of activation in PEA-pre-treated than in vehicle-pre-treated infected animals. Similarly, PEA normalized increased microglial activation in neurodegenerative and neuropathic animal models supporting its neuroprotective properties (34, 35).

One limitation of our study is the use of a single pathogen as representative of bacterial meningitis. We chose to evaluate the effect of PEA in *E. coli* K1 meningitis, because this is one of the predominant causative agents of meningitis in elderly persons (4, 5) and at present no vaccine is available. Further studies are needed to address, whether PEA also mediates protective immune responses in meningitis caused by other bacteria.

Our findings support the therapeutic potential of PEA in the clinical setting as an immunomodulator. PEA could be used as a dietary complement to reduce the number of infections, broaden the potential therapeutic window for the successful use of antibiotics, counteract detrimental inflammation, and improve outcomes of elderly persons upon infection. In our experimental setting, comprising of an infection with a concomitant excessive inflammatory response, PEA promoted bacterial clearance and alleviated the associated inflammatory response, extending the survival of old animals without additional weight loss. PEA acted on the host immune response at three levels: (i) systemic pro-inflammatory cytokine/chemokine signaling, (ii) systemic production of AA and 20-HETE, and (iii) the step-wise activation of microglia.

The mechanisms responsible for the anti-inflammatory and neuroprotective effects of PEA remain unclear. PEA exerts the so-called “entourage effect” (56): an increase in PEA levels could enhance the physiological effects of AEA and 2-AG by preventing their enzymatic-mediated hydrolysis. PEA has been shown to act on several targets, including the transient receptor potential vanilloid type-1 (TRPV1), the peroxisome proliferator-activated receptor-alpha (PPAR-α) and the orphan G-protein coupled receptor GPR55 (57, 58). Another mechanism proposed to explain PEA action is the downregulation of mast cell (MC) activation. MC are important effectors not only in acute/chronic inflammation but also in infection, being capable of modulating the host immune response (59, 60). It is believed that PEA-related therapeutic effects might not be mediated by one exclusive mechanism of action but instead by the synergistic effect on several receptors (44).

Marketed PEA formulations for human use include capsules and PEA as a cream to treat atopic eczema. New formulations with micronized and ultra-micronized PEA are available for oral therapy and have shown improved bioavailability and efficacy compared to naïve PEA (61). In geriatric patients, the efficacy of ultra-micronized PEA (as capsules of 600 mg) is currently under investigation for treatment of chronic pain (62). Given the excellent tolerance and the fact that no adverse effects have been reported after PEA administration (30, 31, 45), a clinical trial should examine whether the use of PEA as a prophylactic agent can reduce the risk of CNS or systemic infections in the immunocompromised geriatric patient. This strategy could additionally help to limit the exposure of the aged population to antimicrobial agents contributing to antimicrobial stewardship, especially relevant in LTCFs (63). When prophylactic PEA cannot prevent an infection, it may be useful to prolong the therapeutic window, in which antibiotic therapy can rescue the aged patient.

## Data availability

Datasets are available on request: The raw data supporting the conclusions of this manuscript will be made available by the corresponding author SR, without undue reservation, to any qualified researcher.

## Ethics statement

All protocols regarding animal housing, welfare and experimentation were reviewed and approved by the local and regional Animal Care Committees at the University Hospital of Göttingen and at the government of Lower Saxony, respectively (Ethics number 14/1553). All animal procedures were carried out in accordance with the laws governing the use of animals for research in Germany (*Tierschutzgesetz*), the European Community regulations (Directive 2010/63/EU), and ARRIVE guidelines.

## Author contributions

RN and SR designed the study. EH and SR performed the animal studies and histological evaluation. JS performed the *in-vitro* growth curves. LB designed and coordinated the lipid profiling. JP performed the lipid extraction and analyses. BL contributed to results interpretation. DM, SR, EH, and RN performed the statistical analyses. SR, EH, DM, LB, and RN interpreted the results. All authors drafted the manuscript and critically revised and approved the final version of the manuscript.

### Conflict of interest statement

DM founded the statistical consultancy mzBiostatistics to provide expert statistical counsel to the research community for high-quality data analysis and data interpretation. DM is the sole proprietor of mzBiostatistics and completely independent of other companies and universities. The remaining authors declare that the research was conducted in the absence of any commercial or financial relationships that could be construed as a potential conflict of interest.
